# Immune landscape of muscle-invasive bladder cancer: role of TIGIT, LAG-3, and PD-L1

**DOI:** 10.3389/fimmu.2026.1777409

**Published:** 2026-03-17

**Authors:** Matej Knežević, Igor Tomašković, Jure Murgić, Borna Vrhovec, Leo Dumbović, Val Vrbić, Sebastijan Trifunić, Milan Milošević, Monika Ulamec

**Affiliations:** 1 Clinical Department of Urology, University Hospital Center “Sestre milosrdnice”, Zagreb, Croatia; 2 School of Medicine, Croatian Catholic University, Zagreb, Croatia; 3 School of Medicine, “Josip Juraj Strossmayer” University, Osijek, Croatia; 4 Clinical Department of Oncology and Nuclear Medicine, University Hospital Center “Sestre milosrdnice”, Zagreb, Croatia; 5 School of Medicine, University of Zagreb, Zagreb, Croatia; 6 Clinical Department of Pathology and Cytology “Ljudevit Jurak”, University Hospital Center “Sestre milosrdnice”, Zagreb, Croatia

**Keywords:** biomarkers, LAG-3, molecular subtypes, muscle-invasive bladder cancer, PD-L1, TIGIT, tumor microenvironment

## Abstract

**Introduction:**

Muscle-invasive bladder cancer (MIBC) is an aggressive disease that typically requires multimodal treatment. Recently, immunotherapy strategies targeting the tumor microenvironment (TME) have reshaped the therapeutic approach for MIBC. Our study explores the expression of immune checkpoint biomarkers TIGIT, LAG-3, and PD-L1 across molecular subtypes of MIBC.

**Methods:**

Immunohistochemical analysis was performed on archival tumor samples from 62 patients, evenly split between luminal and basal molecular MIBC subtypes.

**Results:**

The findings revealed that luminal MIBC patients with high stromal LAG-3 expression had significantly worse survival outcomes compared to those with the basal subtype, establishing LAG-3 as an independent prognostic marker of poor survival in luminal MIBC. In the basal subtype, LAG-3 was more frequently expressed in the stroma than in the luminal subtype. TIGIT expression was consistently detected in both stromal cells and epithelial tumor cells, highlighting its potential as an immunotherapy target. PD-L1 expression showed a positive correlation with both LAG-3 and TIGIT levels in the stroma across both subtypes. The strong immune activity of these ligands underscores their potential as targets for immunotherapy.

**Discussion:**

These results may enhance the understanding of MIBC’s immune landscape and help identify patient subgroups who could benefit from immune-based treatments. However, validation in larger patient cohorts is needed to confirm the clinical relevance of these biomarkers.

## 1 Introduction

Bladder cancer (BC) is a complex disease associated with a high morbidity and mortality rate. It accounts for 3% of all cancers globally, with a higher incidence in developed countries. The disease predominantly affects older adults, with a median age at diagnosis of around 65 years and shows a male predominance with an incidence of 9.5 per 100,000 in men compared to 2.4 in women. The mortality rate is 1.9 per 100,000 (1). Five-year survival is 75%, and in metastatic disease up to 5%. The primary risk factor remains cigarette smoking, contributing to over 50% of cases, alongside occupational exposure to aromatic amines and chronic inflammation (2).

Histologically, over 90% of BC cases are urothelial carcinomas (UC). Among them, approximately 85% are initially diagnosed as non-muscle-invasive bladder cancer (NMIBC), confined to mucosa or submucosa. These tumors are typically managed with transurethral resection of bladder tumor (TURBT) followed by intravesical therapy, such as Bacillus Calmette-Guérin (BCG), which serves as an immunomodulator to reduce recurrence and progression (3, 4). In contrast, muscle-invasive bladder cancer (MIBC) accounts for the remaining 15% and is associated with a significantly worse prognosis, five-year survival of about 50%, and a high risk of progression and metastasis (5, 6). Accordingly, MIBC requires early detection and adequate active treatment.

The standard treatment for MIBC includes radical cystectomy (RC), often preceded by cisplatin-based neoadjuvant chemotherapy (NAC) to address micrometastatic disease (7). However, only 25–30% of patients are suitable candidates for NAC due to impaired renal function, frailty, or comorbidities. Moreover, despite aggressive treatment, approximately half of MIBC patients develop disease recurrence or metastasis within one year after RC, highlighting the urgent need for novel therapeutic strategies (8–10).

In recent years, immune checkpoint inhibitors (ICIs) have transformed the management of advanced and metastatic BC. By targeting co-inhibitory pathways, most notably the programmed death-1 (PD-1) receptor and its ligand PD-L1, these therapies restore cytotoxic T-cell function and enhance antitumor responses. Agents such as atezolizumab and pembrolizumab are approved for metastatic UC in patients who have progressed following or are ineligible for cisplatin-based chemotherapy. While ICIs can achieve durable responses in a subset of patients, overall response rates remain modest, underscoring the need for robust predictive biomarkers (11).

Genomic alterations represent the primary drivers of BC carcinogenesis. MIBC is characterized by a high mutational burden, particularly in genes regulating the cell cycle (12). Multiple molecular classification systems based on global messenger RNA (mRNA) expression have been proposed, with luminal and basal subtypes emerging as the predominant categories. Luminal MIBC typically exhibits resistance to chemotherapy, whereas basal MIBC demonstrates squamous differentiation, aggressive clinical behavior, and a comparatively favorable response to chemotherapy (13, 14).

Within this molecular framework, the TME has gained prominence as a central mediator of oncogenesis and immune regulation, as well as a therapeutic target for ICIs (15, 16). Tumor-infiltrating immune cells, including regulatory T cells, macrophages, mast cells, and B cells, play a pivotal role in modulating antitumor immunity in MIBC (17). Advances in molecular and immunoprofiling have underscored their potential to redefine treatment paradigms.

Although immuno-oncology research is developing in full swing, studies focused on the expression of immune checkpoints in MIBC are still rare (18). The effectiveness of current ICI has stimulated interest in finding potential new therapeutic targets - control points. In recent years, research attention has expanded beyond PD-L1 to additional immune checkpoint ligands, notably T-cell immunoreceptor with Ig and ITIM domain (TIGIT) and lymphocyte activation gene 3 (LAG-3), both expressed on T cells and implicated in antitumor immune regulation (19–22).

TIGIT is a co-inhibitory receptor present within the TME that suppresses protective immune responses mediated by CD8^+^ T cells and natural killer (NK) cells (23). Preclinical studies have demonstrated that TIGIT blockade restores T-cell function and enhances antitumor immunity in acute myeloid leukemia, colorectal cancer, and melanoma (24–26). Several anti-TIGIT monoclonal antibodies are currently under clinical development, with experimental evidence indicating a synergistic effect when combined with anti-PD-L1 therapy (27, 28). Despite these advances, TIGIT expression and clinical relevance in MIBC remain insufficiently characterized. LAG-3 is a transmembrane protein expressed on activated T cells and NK cells (29). Experimental data indicate that LAG-3 signaling contributes to CD8^+^ T-cell exhaustion, whereas LAG-3 blockade can reinvigorate T-cell activity and potentiate antitumor immunity. Similar to TIGIT, LAG-3 inhibition may act synergistically with PD-L1 blockade (19, 30). However, the prognostic implications of LAG-3^+^ cell infiltration vary among cancer types: in breast and ovarian cancers, high LAG-3^+^ cell density has been associated with improved prognosis and sustained antitumor T-cell immunity, whereas in lung cancer it correlates with poor survival, likely reflecting an immunosuppressive tumor milieu (31–33).

PD-L1, TIGIT, and LAG-3 represent actionable targets for immunotherapy; however, their comprehensive significance within the immune context of MIBC and their interplay with molecular MIBC subtypes remain undefined. Given that ligand expression may differ between luminal and basal subtypes, these markers hold potential as both prognostic and predictive biomarkers, paving the way for more individualized therapeutic strategies. While immune checkpoint expression has been investigated in bladder cancer, most prior studies have evaluated PD-L1, LAG-3, or TIGIT expression without systematic stratification according to molecular subtype and often without separate analysis of tumor epithelial and stromal compartments (34–37). Furthermore, data on stromal LAG-3 expression in MIBC remain limited, and its potential subtype-specific prognostic relevance has not been clearly defined. Given the recognized biological differences between luminal and basal MIBC, integrating molecular subtype classification with compartment-specific immune profiling may provide more refined insights into the TME and its clinical implications. Therefore, the present study was designed to assess TIGIT, LAG-3, and PD-L1 expression in both tumoral epithelial and stromal compartments, stratified by luminal and basal molecular subtypes, and to evaluate their association with survival outcomes.

## 2 Materials and methods

### 2.1 Patients

A cross-sectional retrospective study utilized archival MIBC samples from the “Ljudevit Jurak” Institute of Pathology and Cytology at the “Sestre milosrdnice” University Hospital Center in Zagreb, Croatia. The samples were obtained by a random selection of 62 patients from a cohort of 185 patients who underwent TURBT for the diagnosis and treatment of BC, in the period from 01.01.2016. until 31.12.2021. All tumor specimens included in this study were obtained at the time of initial surgical management (TURBT), and all patients were therapy-naive at the time of tissue collection. None had received systemic chemotherapy, immunotherapy, or radiotherapy prior to sampling. The selection ensured balanced representation of the luminal and basal molecular subtypes. Clinical and pathological characteristics of the patient were monitored through one postoperative year. Clinical data were extracted from patient records, while histological data were obtained from the institutional databases.

### 2.2 Experimental procedures

**Figure 1:**
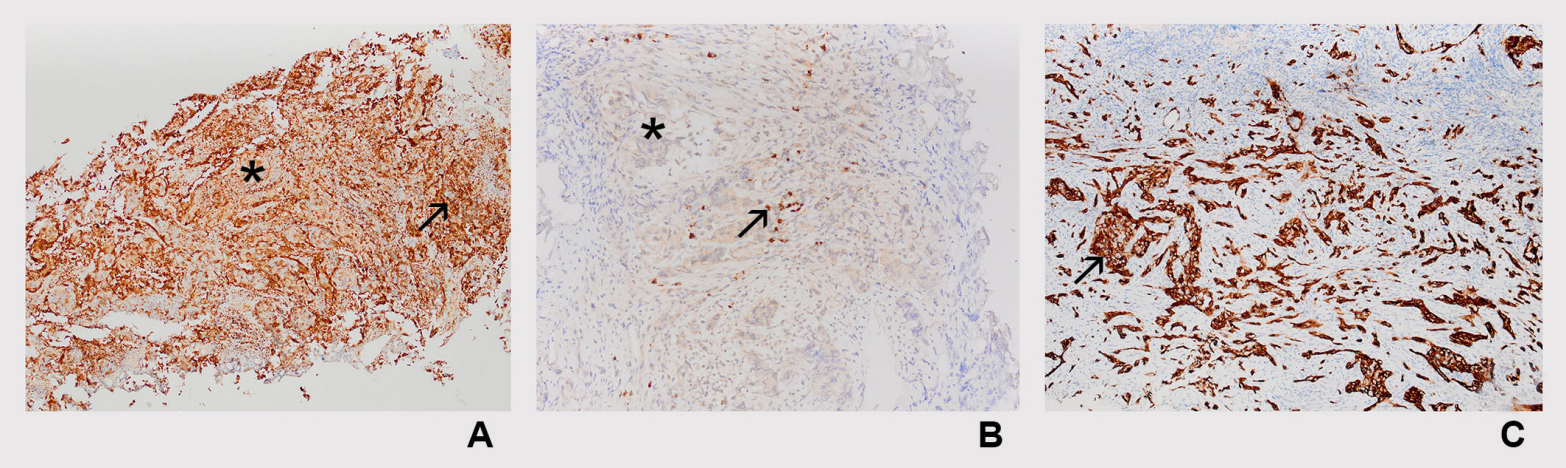
Representative immunohistochemical staining of immune checkpoint markers. Expression was evaluated separately in tumor epithelial cells (epithelial tumoral compartment) and tumor-associated stromal cells (stromal compartment), with positivity defined as staining in ≥5% of cells within the respective compartment: **(A)** reaction to TIGIT, moderately strong epithelial (tumoral) staining intensity (asterisk), with strong stromal cell staining intensity (arrow) (TIGITx100); **(B)** reaction to LAG-3, mild epithelial (tumoral) cell staining intensity (asterisk), with moderate stromal cell staining intensity (arrow) (LAG-3x200); **(C)** reaction to PD-L1, strong epithelial (tumoral) staining intensity (arrow), without stromal cell reaction (PD-L1x100).

Formalin fixed paraffin-embedded (FFPE) tissue blocks from TURBT specimens were processed using standard histological protocols including tissue fixation in 10% buffered formalin immediately after resection, dehydration in an ascending series of alcohol, embedding in paraffin blocks, sectioning at a thickness of 5 μm, deparaffinization in xylene and staining with the standard hemalaun eosin (HE) method. Each case was reviewed on five HE-stained sections to confirm MIBC diagnosis and select representative blocks for immunohistochemistry (IHC). The analysis was performed on a paraffin block containing a minimum of 10 TURBT tissue samples, within which cancer tissue made up at least 50% of the samples. In addition to tumor tissue, the samples also contained normal mucosa, with preserved urothelium on the surface. Molecular subtyping was performed via IHC for CK5/6 (DAKO IR78061-2) and GATA3 (Ventana L50-823) (13, 38). Skin tissue for CK 5/6 and low-grade urothelial carcinoma tissue for GATA3 were used as positive controls. Luminal subtype was defined by ≥70% GATA3-positive tumor cells and CK5/6 negativity; basal subtype by ≥70% CK5/6 positivity and GATA3 negativity. Tumors not fitting these criteria were excluded. The following antibodies were used for IHC analysis of the studied ligands: anti-TIGIT (ABCAM BLR047F), LAG-3 (Sigma-Aldrich HPA013967) and PD-L1 (Ventana SP142). SP142 PD-L1 antibody clone has known variability in sensitivity compared with other PD-L1 clones, which may influence comparability across studies. Regarding the limitations of the SP142 PD-L1 antibody clone, it is important to note that this assay is primarily validated and routinely used for the assessment of PD-L1 expression in stromal cells and for the determination of the immune cell score (ICS). It is not specifically designed for the evaluation of PD-L1 expression in tumor epithelial cells, which may limit direct comparability with studies using other antibody clones optimized for tumor cell staining. IHC was performed using the indirect ABC technique, with LSAB (from labeled streptavidin - biotin) method as the visualization system on a Dako TechMate TM and Ventana automated IHC staining machine using the Microwave Streptavidin ImmunoPeroxidase (MSIP) protocol. Recommended positive controls were used: tonsil (for PD-L1), lymph node (for TIGIT and LAG-3). Negative process controls were performed according to the manufacturer’s recommendations using tonsil tissue. In each staining run, the primary antibody was omitted and replaced with non-immune serum under identical conditions, confirming the absence of non-specific background staining. The slides were evaluated independently by two observers: an experienced pathologist and a PhD student under mentor supervision, with any discrepancies resolved by consensus. Staining patterns were assessed both at low (40x) and high (200x/400x) magnification. For the purpose of analysis, immune checkpoint expression was assessed separately in two predefined compartments: (a) the epithelial tumoral compartment, defined as malignant epithelial tumor cells, and (b) the stromal compartment, defined as non-epithelial tumor-associated stromal cells within the tumor microenvironment, including immune cells, where inflammatory response occurs. IHC reactions for all antibodies were assessed semiquantitatively by determining both the percentage of positive epithelial tumor cells and the percentage of positive stromal cells. For TIGIT and LAG-3, positive staining in epithelial cells was identified as a brown signal localized to the cytoplasm and cell membrane (**Figures 1A, B**). PD-L1 positivity in epithelial cells was defined by brown membranous staining (**Figure 1C**). Staining intensity was graded as follows: [1] weak – very faint, barely perceptible brown staining; [2] moderate – clearly visible brown staining; and [3] strong – dark brown staining. In addition to intensity, the absolute percentage of positively stained tumor cells in the examined section was recorded. Stromal cells (lymphocytes, plasma cells) in both intra- and peritumoral stroma were similarly evaluated for ligand expression. Staining patterns most frequently consisted of small lymphocyte clusters, and less commonly diffuse infiltrates. A 5% cutoff was applied to define positivity. Adjacent non-tumor urothelium was analyzed as a reference. A 5% cutoff was selected based on both practical and literature-based reasons. For LAG-3 and TIGIT, currently there is no standardized scoring system in MIBC, and previous IHC studies across different tumor types have used different range of thresholds and analytical approaches (39, 40). In contrast, for PD-L1 there are established clinical cutoffs used in therapeutic decision-making, typically around 10% for stromal immune cells and 5% for tumor epithelial cells (33). Considering the exploratory nature of this study and the absence of standardized scoring systems for LAG-3 and TIGIT in MIBC, the 5% threshold was applied uniformly across all markers to ensure comparability.

### 2.3 Ethical statement

The institutional ethical committee reviewed and approved the study. Patient consents were obtained during the diagnostic and therapeutic procedures.

### 2.4 Statistical analysis

The distribution of numerical data was evaluated for normality using the Smirnov-Kolmogorov test, and non-parametric tests were used based on the results. Quantitative data is presented through medians and interquartile ranges. Categorical data are presented through absolute frequencies and corresponding shares. Differences in quantitative values ​​were analyzed by the Mann-Whitney U test for differences related to the comparison of continuous values ​​between two groups. Differences in categorical variables were analyzed by Fisher’s exact test in cases of 2x2 format comparisons, or by Fisher-Freeman-Halton’s exact test when 2x3 or larger table formats were compared. Correlation analysis was performed using Kendall’s tau_b correlation coefficients. Survival analysis is shown by the Kaplan-Meier curve with the corresponding log-rank test. A multivariate Cox proportional hazards regression analysis was performed in addition to the Kaplan–Meier survival analysis. However, due to the relatively limited sample size and the number of survival events, the multivariate model did not yield statistically significant or clinically meaningful independent predictors. Given these constraints and the exploratory nature of the study, we chose not to include the full Cox model results in the original version of the manuscript to avoid overinterpretation of statistically unstable estimates. All P values ​​less than 0.05 were considered statistically significant. The analysis used IBM SPSS Statistics for Windows, version 29.0.1.

## 3 Results

The IHC expression of the biomarkers TIGIT, LAG-3 and PD-L1 was investigated in 62 samples of MIBC. The average age of the patients included in the study was 70 years with a median of 67 years (ranging from 49 to 85). The distribution of men to women within the group was 75.81% to 24.19%, which corresponds to the expected epidemiological gender distribution for BC. The general status of the patients according to the ECOG scale (from: English Eastern Cooperative Oncology Group) was completely normal in almost half of the patients. The most common comorbid conditions included arterial hypertension, heart disease, diabetes and urinary tract infections. Considering the patient habits, 66.13% were smokers and 33.87% were non-smokers. Analyzing the characteristics of BC, the disease was unifocal in as many as 90% of the patients. The initial assessment of MIBC disease extent in 61.29% of patients corresponded to T2 stage without positive lymph nodes. The initial TURBT was significant for this study, as well as the pathohistological and IHC analysis of samples obtained from the primary resection. However, all patients underwent additional treatment in the subsequent course. This was further individually tailored treatment, which included radical cystectomy in 53.23% of patients. NAC was administered at 24.19%, and adjuvant radiotherapy in only 9.68% of patients. The low rate of chemotherapy administration corresponds to the already mentioned small number of patients suitable for cisplatin administration, which leaves the possibility of immunotherapy as a treatment option. Primary radiotherapy was received by 22.58% of patients, and 12.9% were included in the trimodal approach for bladder-preserving treatment. Despite all therapeutic options, BC is prone to relapse. Local relapses were detected in 32.26%, regional in 29.03% of cases, while systemic disease was present in 41.94% of patients. Overall survival in our study was 56.45%. Of the 62 patients, 27 (43.55%) died. No statistically significant differences in categorical clinical variables were observed between the study groups.

### 3.1 TIGIT expression

There were no statistically significant differences regarding the TIGIT intensity in stromal tissue between the basal and luminal molecular subtypes of MIBC (P = 0.421). However, in all examined samples of both subtypes, the intensity of the positive reaction in stromal cells was present, at least moderately, and in a noticeable number of cases, also strongly. Analysis of the TIGIT expression in the stroma showed that the proportion of positive cells ranged from 5% to 70%. A positive reaction to TIGIT was observed in almost all cases, i.e. in 87.10% of basal and 93.55% of luminal MIBC. In a small number of negative reactions (with a proportion of < 5% positive stromal cells), the presence of TIGIT at a lower level of expression was also observed. Such positive expressions in most cases may suggest TIGIT as a potential target site for immunotherapy. There were no statistically significant differences in TIGIT expression in basal versus luminal MIBC (P = 0.390). There were no statistically significant differences of the TIGIT intensity in the epithelium between the basal and luminal MIBC (P = 0.411). In the case of a positive reaction in the samples examined, it was mostly moderate or weak intensity. Analysis of TIGIT expression in the epithelium for basal and luminal molecular subtypes indicated that TIGIT was positive in a significant number of cases, namely present in 35.48% of basal and 54.84% of luminal MIBC cases. There were no statistically significant differences between the basal and luminal molecular subtypes (P = 0.126). The range of positive reactions was between 5 - 50%.

### 3.2 LAG-3 expression

The differences in IHC LAG-3 intensity in the stroma between the basal and luminal molecular subtypes of MIBC were observed. In case of a positive reaction, moderate intensity was found. Weak and strong intensity were not detected in any case. Statistically significant differences were found between the basal and luminal molecular subtypes of MIBC (P = 0.002). In the basal molecular subtype, moderate intensity was the most common (80.6%), while in the luminal subtype, a negative finding was the most common (58.1%). Regarding the LAG-3 expression, the proportion of positive cells in the stroma ranged from a minimum of 5% to a maximum of 10%. In the basal subtype, a statistically significantly higher positive expression was observed compared to the luminal subtype: 19.4% versus 3.2% (P = 0.045). Intensity analysis of LAG-3 in the epithelium found no statistically significant differences between the molecular subtypes (P = 0.356). LAG-3 expression in the epithelium showed negative reaction in all cases through our cohort.

### 3.3 PD-L1 expression

The IHC PD-L1 expression in epithelial tumor cells was recorded in 45.16% of basal and 32.26% of luminal MIBC. There were no statistically significant differences in PD-L1 expression between the basal and luminal MIBC. PD-L1 positivity ranged from 5% to 20%. In 9.3% of all MIBC cases, a distinct positive reaction was observed in the epithelial tumor cells (>10% of positive cells), mainly in basal subtypes.

### 3.4 Quantitative and correlation analyses

**Figure 2:**
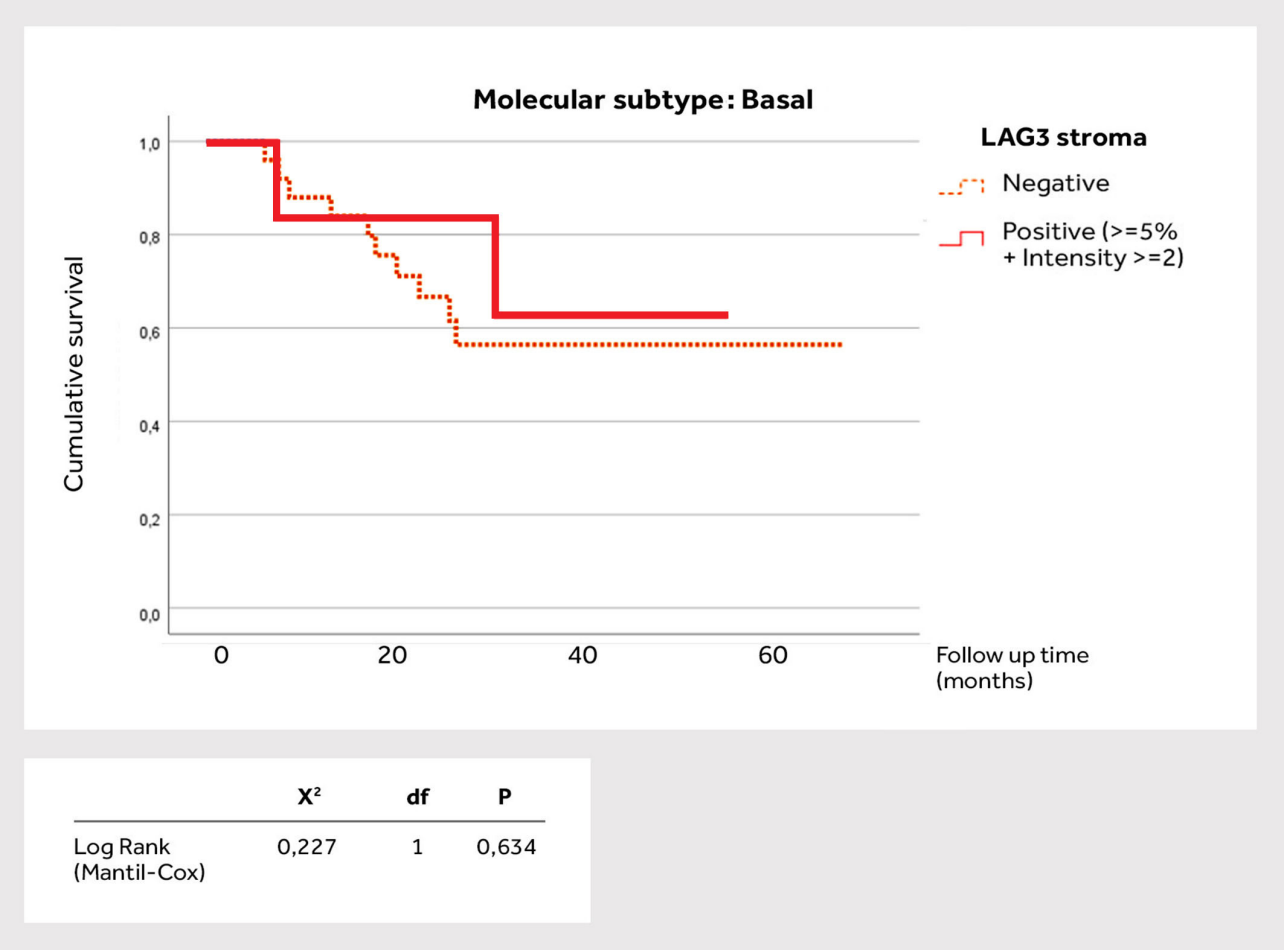
Survival analysis according to LAG-3 positivity in the stroma of basal muscle-invasive bladder cancer subtype.

**Figure 3:**
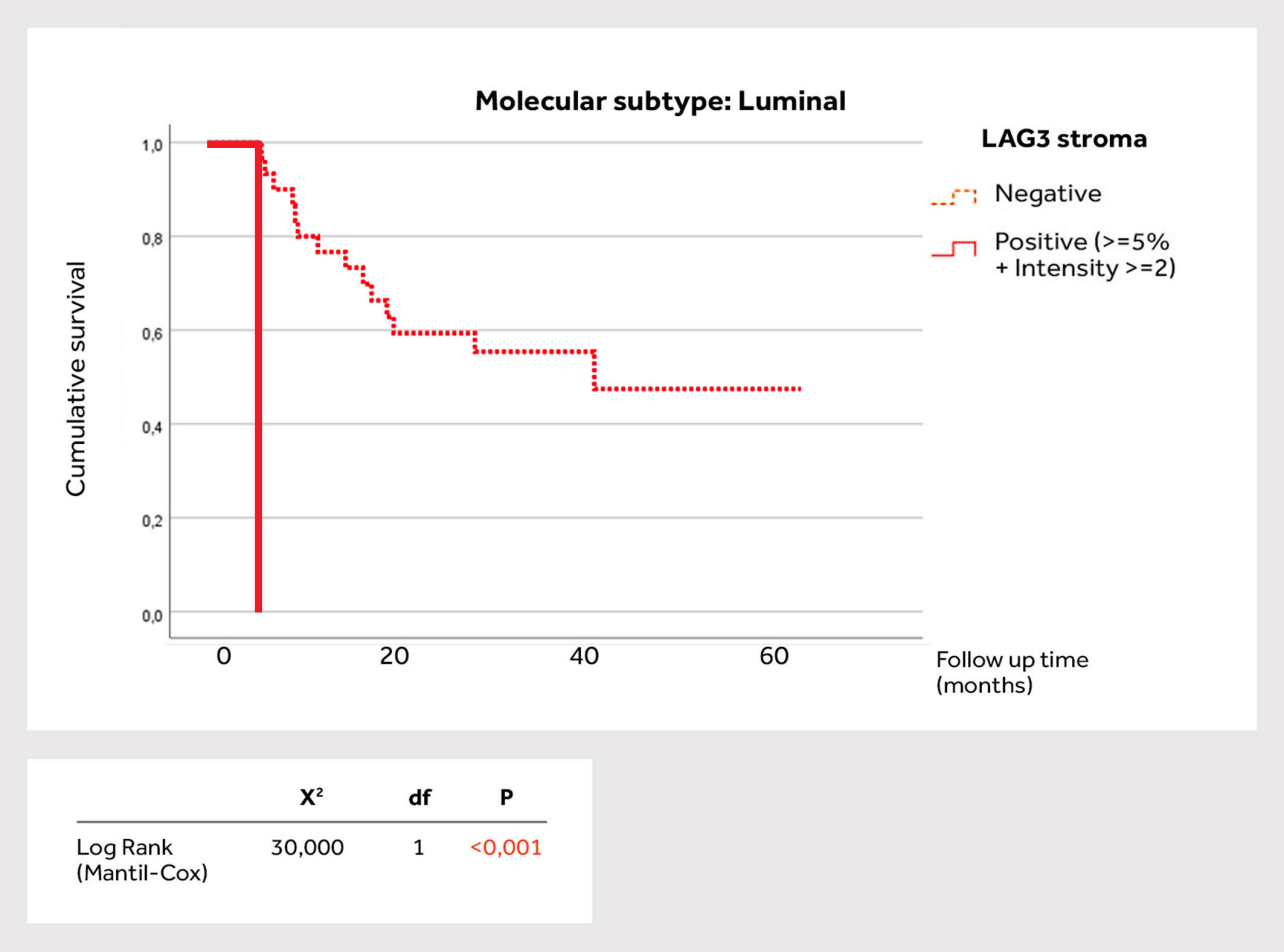
Survival analysis according to LAG-3 positivity in the stroma of luminal muscle-invasive bladder cancer subtype.

Stromal LAG-3 expression was significantly higher in the basal subtype (P = 0.002). There was a significant positive correlation between PD-L1 expression and LAG-3 intensity in the stroma (P = 0.005), as well as TIGIT expression in the stroma (P = 0.03). There were also significant positive correlations between LAG-3 intensity in the stroma and TIGIT expression in the stroma (P = 0.021), as well as correlations between LAG-3 expression with LAG-3 intensity in the epithelium (P = 0.028). There were significant positive correlations between TIGIT intensity and LAG-3 intensity and expression in the epithelium (P = 0.046). Survival for individual molecular MIBC subtype was also analyzed in relation to the biomarker positivity in the stroma, especially LAG-3 findings. The basal MIBC (**Figure 2**) showed no significant association with survival (P = 0.634). On the other hand, in the luminal subtype LAG-3 positivity was significantly associated with reduced survival (P<0.001), where the average survival time of positive LAG-3 patients was 5.26 months, while the negative patients averaged 38.23 months (**Figure 3**).

## 4 Discussion

Muscle-invasive bladder cancer (MIBC) is a biologically aggressive and clinically challenging entity within the spectrum of BC. Driven by DNA mutations affecting cell cycle regulation, MIBC has been increasingly characterized through molecular subtyping, most notably into luminal and basal categories. These subtypes differ not only in morphology and marker expression (luminal subtypes expressing GATA3 and basal expressing CK5/6), but also in clinical behavior, therapeutic responsiveness, and prognosis. Luminal tumors often exhibit resistance to chemotherapy, whereas basal tumors, though more aggressive, tend to respond better to systemic treatments (41).

Recent advances have highlighted the importance of TME as a dynamic and critical space of tumor progression and treatment resistance (42). The TME comprises immune cells, stromal components, and signaling molecules that interact with tumor cells to shape immune responses. This realization has shifted research focus toward ICIs, which aim to reverse immune suppression by targeting key inhibitory molecules like PD-L1 (43). While PD-L1 has become the most widely studied and clinically used biomarker for ICI therapy, its utility is limited. Although the expressed PD-L1 value is considered a prerequisite for the use of ICI, it is not a reliable criterion for the selection of patients who will benefit from ICI. The limitations of PD-L1 as a predictive marker include variability in expression, technical inconsistencies, and different antibody clones, which can bring additional heterogeneity of data and results (44, 45).

Given these challenges, attention has shifted toward novel immune checkpoint molecules such as LAG-3 and TIGIT, which may provide alternative or complementary pathways for immune modulation. LAG-3 is a transmembrane protein expressed on activated T-cells and NK cells, known to suppress T-cell proliferation and cytokine secretion. TIGIT, similarly, is an inhibitory receptor found on T-cells and NK cells, functioning through its ligands to reduce cytotoxic activity. Both are being actively explored in clinical trials across various malignancies (20, 30).

Our study sought to evaluate the IHC expression of PD-L1, LAG-3, and TIGIT in MIBC, examining their presence in both epithelial tumor cells and stromal cells, and their correlation with molecular subtypes and clinical outcomes. In a cohort of 62 patients (31 luminal, 31 basal), PD-L1 was more frequently expressed in basal tumors (45.16%) than luminal (32.26%), consistent with prior studies suggesting PD-L1 upregulation in more aggressive tumor phenotypes (46). LAG-3 expression was largely restricted to stromal cells and significantly associated with worse survival in luminal MIBC. None of the luminal tumors showed LAG-3 positivity in epithelial cells, but those with LAG-3-positive stromal infiltrates had dramatically lower survival (mean 5.26 months vs. 38.23 months, p<0.001). These findings position LAG-3 as a potential prognostic biomarker in luminal MIBC and a possible target for therapeutic intervention. TIGIT expression was near-universal in stroma and frequently found in epithelial tumor cells, with no significant differences between subtypes. However, the high prevalence and intensity of TIGIT staining supports its relevance as a candidate for future immunotherapeutic strategies.

The subtype-specific prognostic effect of stromal LAG-3 observed in our cohort raises important biological considerations. Luminal MIBC is generally characterized by a more differentiated phenotype and lower baseline immune infiltration compared to basal tumors, which are typically more inflamed and immunologically active. In this context, the presence of LAG-3–positive stromal immune cells in luminal tumors may reflect the emergence of an acquired immunosuppressive microenvironment, representing a shift toward T-cell exhaustion and ineffective antitumor immunity. Conversely, basal tumors, despite demonstrating higher stromal LAG-3 expression overall, may already harbor a highly inflamed TME in which additional checkpoint expression does not further stratify prognosis. This difference may explain why LAG-3 carries prognostic significance specifically in luminal MIBC. These interpretations are hypothesis-generating and require further validation.

Our findings align with a growing body of literature and suggest that immune evasion in MIBC is mediated by a network of immune checkpoints beyond PD-L1 alone (47). Previous studies have documented the prognostic impact of LAG-3 in several cancers, including melanoma, glioma, lung, and breast cancer. In MIBC, Zeng et al. (2021) reported that stromal LAG-3 expression correlated with dysfunctional CD8+ T-cells and poor response to NAC, findings echoed in our study (21). For TIGIT, its role in immune suppression is increasingly recognized (48). Liu et al. (2022) found that high TIGIT+ CD8+ T-cell infiltration predicted poor outcomes and chemoresistance in MIBC (49). Our observation of widespread TIGIT positivity in both epithelial and stromal compartments reinforces its potential as a universal target across molecular subtypes.

Importantly, our study also demonstrated significant positive correlations between the expressions of PD-L1, LAG-3, and TIGIT. This suggests a concerted expression of multiple immune checkpoints in the same tumors, supporting the hypothesis that combination checkpoint blockade may offer superior clinical benefits compared to monotherapies (50). Preclinical studies have shown synergistic effects between anti-PD-1/PD-L1 and anti-LAG-3 or anti-TIGIT therapies, although clinical validation remains pending (51).

There are several limitations of the study. The study’s single-center, retrospective design limits the generalizability of our findings. Due to the retrospective and observational design, findings should be interpreted as associative rather than causal. The use of archival formalin-fixed paraffin-embedded (FFPE) tissue restricts molecular analyses, and our classification into luminal and basal types did not include further subclassifications, which may harbor additional prognostic and predictive information (52). The cutoff for IHC positivity was 5% for all markers and it was selected to balance sensitivity for detecting biologically relevant immune infiltration and consistency with prior literature. We acknowledge that different cutoffs may influence classification and potentially affect statistical associations. Additionally, inter-assay variability and the lack of standardized scoring criteria for LAG-3 and TIGIT limit the reproducibility of results. The IHC-based assessment, while practical, may not fully capture the dynamic and spatial complexity of the immune TME (53). Prospective trials incorporating genomic and transcriptomic profiling could elucidate the mechanisms driving differential expression of immune checkpoints. Moreover, studies evaluating the predictive value of these markers in patients receiving ICI therapy would be crucial for clinical translation. There is also a need to standardize staining protocols and scoring systems for emerging checkpoints like LAG-3 and TIGIT, to ensure comparability across studies. Integrating immune marker profiling into routine diagnostic workflows may aid in patient stratification and personalized treatment planning. Finaly, a multivariate Cox analysis was conducted in addition to the Kaplan–Meier survival analysis, but no independent predictors were identified, likely due to limited statistical power; thus, the results should be interpreted as exploratory and validated in larger studies.

The observed association between increased stromal LAG-3 expression and reduced overall survival in luminal MIBC may have potential translational implications. Although exploratory, these findings suggest that immune profiling beyond PD-L1 alone could contribute to more refined patient stratification within molecular subtypes. In particular, the identification of a subgroup of luminal tumors characterized by higher stromal LAG-3 expression may reflect a distinct immunological TME with potential therapeutic relevance. Given the emerging clinical development of LAG-3 inhibitors and combination immune checkpoint blockade strategies, our results support further investigation into whether luminal MIBC patients with elevated stromal LAG-3 expression could represent a biologically defined subgroup that may benefit from tailored immunotherapeutic approaches (54). Prospective validation in larger cohorts and in the context of treatment response data will be necessary to determine the clinical utility of this marker. Previous studies have reported variable expression of LAG-3, TIGIT, and PD-L1 in bladder cancer and other solid tumors; however, most analyses have not incorporated molecular subtype stratification or compartment-specific assessment (31, 33, 55). For example, prior investigations in urothelial carcinoma have demonstrated LAG-3 expression predominantly in tumor-infiltrating lymphocytes, often associating higher immune checkpoint expression with an inflamed TME, but without distinguishing luminal and basal subtypes or separately analyzing stromal versus epithelial compartments (29). Similarly, studies evaluating TIGIT and PD-L1 expression in bladder cancer have largely focused on overall expression rates or therapeutic predictive value, rather than subtype-specific prognostic implications (20). In contrast, our study integrates molecular subtype classification with compartment-resolved immune checkpoint assessment and survival analysis. Notably, we observed that increased stromal LAG-3 expression was associated with reduced overall survival specifically in luminal MIBC, while this association was not evident in basal tumors. This subtype-restricted prognostic effect suggests that immune checkpoint biology may differ fundamentally between luminal and basal MIBC, reflecting distinct TME interactions. To our knowledge, data specifically addressing stromal LAG-3 expression in MIBC in a subtype-stratified context remain scarce (56). Therefore, our findings provide novel evidence that integrating molecular subtype with stromal immune checkpoint profiling may uncover clinically relevant heterogeneity not captured by analyses that evaluate immune markers in aggregate. These results extend prior reports of LAG-3 expression in solid tumors, where its presence has variably been associated with immune exhaustion, tumor aggressiveness, or response to immunotherapy, but rarely examined within a molecularly defined urothelial cancer framework (57). Future studies incorporating larger cohorts and treatment response data will be necessary to validate the prognostic and potential predictive relevance of stromal LAG-3 in luminal MIBC.

This study adds to the growing body of evidence that immune checkpoint expression in MIBC is both heterogeneous and molecular subtype–specific. Our results identify LAG-3 as a potential prognostic biomarker in luminal MIBC and demonstrate that TIGIT is widely expressed across both subtypes. The frequent co-expression of PD-L1, LAG-3, and TIGIT suggests that combinatorial immune checkpoint blockade may offer therapeutic benefit. These findings support the incorporation of immune profiling into molecular classification frameworks for MIBC and provide a rationale for developing multi-target immunotherapeutic strategies.

## Data Availability

The raw data supporting the conclusions of this article will be made available by the authors, without undue reservation.

## References

[1] GLOBCANBladder-fact-sheet.pdf. Available online at: https://gco.iarc.fr/today/fact-sheets-cancers (Accessed December 12, 2024).

[2] Saginala K Barsouk A Aluru JS Rawla P Padala SA Barsouk A . Epidemiology of bladder cancer. Med Sci (Basel). (2020) 8:15. doi: 10.3390/medsci8010015, PMID: 32183076 PMC7151633

[3] Babjuk M Burger M Comperat EM Gontero P Mostafid AH Palou J . European association of urology guidelines on nonmuscle-invasive bladder cancer (TaT1 and carcinoma *in situ*) - 2019 update. Eur Urol. (2019) 76:639–57. doi: 10.1016/j.eururo.2019.08.016, PMID: 31443960

[4] Peyton CC Chipollini J Azizi M Kamat AM Gilbert SM Spiess PE . Updates on the use of intravesical therapies for non-muscle invasive bladder cancer: how, when and what. World J Urol. (2019) 37:2017–29. doi: 10.1007/s00345-018-2591-1, PMID: 30535583

[5] Moch H Humphrey PA Ulbright TM Reuter VE . Tumours of the urinary tract. U: World Health Organization classification of tumours of the urinary system and male genital organs. 4th ed. Lyon, France: IARC Press (2016) p. 77–133.

[6] Jordan B Meeks JJ . T1 bladder cancer: current considerations for diagnosis and management. Nat Rev Urol. (2019) 16:23–34. doi: 10.1038/s41585-018-0105-y, PMID: 30323201

[7] Aminoltejari K Black PC . Radical cystectomy: a review of techniques, developments and controversies. Transl Androl Urol. (2020) 9:3073–81. doi: 10.21037/tau.2020.03.23, PMID: 33457280 PMC7807330

[8] Witjes JA Bruins HM Cathomas R Comperat EM Cowan NC Gakis G . European association of urology guidelines on muscle-invasive and metastatic bladder cancer: summary of the 2020 guidelines. Eur Urol. (2021) 79:82–104. doi: 10.1016/j.eururo.2023.08.016, PMID: 32360052

[9] Flaig TW Spiess PE Agarwal N Bangs R Boorjian SA Buyyounouski MK . Bladder cancer, version 3.2020, NCCN clinical practice guidelines in oncology. J Natl Compr Canc Netw. (2020) 18:329–54. doi: 10.6004/jnccn.2020.0011, PMID: 32135513

[10] Koshkin VS Barata PC Rybicki LA Zahoor H Almassi N Redden AM . Feasibility of cisplatin-based neoadjuvant chemotherapy in muscle-invasive bladder cancer patients with diminished renal function. Clin Genitourin Cancer. (2018) 16:e879–92. doi: 10.1016/j.clgc.2018.02.002, PMID: 29576445

[11] Rijnders M de Wit R Boormans JL Lolkema MPJ van der Veldt AAM . Systematic review of immune checkpoint inhibition in urological cancers. Eur Urol. (2017) 72:411–23. doi: 10.1016/j.eururo.2017.06.012, PMID: 28645491

[12] Guo CC Czerniak B . Bladder cancer in the genomic era. Arch Pathol Lab Med. (2019) 143:695–704. doi: 10.5858/arpa.2018-0329-RA, PMID: 30672335

[13] Meeks JJ Al-Ahmadie H Faltas BM Taylor 3JA Flaig TW DeGraff DJ . Genomic heterogeneity in bladder cancer: challenges and possible solutions to improve outcomes. Nat Rev Urol. (2020) 17:259–70. doi: 10.1038/s41585-020-0304-1, PMID: 32235944 PMC7968350

[14] Terlević R Ulamec M Štimac G Murgić J Krušlin B . Molecular classification of muscle-invasive bladder cancer based on a simplified immunohistochemical panel using GATA3, CK5/6 and p16. Biomol Biomed. (2023) 23:968–75. doi: 10.17305/bb.2023.9242, PMID: 37389960 PMC10655881

[15] Fu H Zhu Y Wang Y Liu Z Zhang J Xie H . Identification and validation of stromal immunotype predict survival and benefit from adjuvant chemotherapy in patients with muscle-invasive bladder cancer. Clin Cancer Res. (2018) 24:3069–78. doi: 10.1158/1078-0432.CCR-17-2687, PMID: 29514839

[16] Robertson AG Kim J Al-Ahmadie H Bellmunt J Guo G Cherniack AD . Comprehensive molecular characterization of muscle-invasive bladder cancer. Cell. (2017) 171:540–56.e25. doi: 10.1016/j.cell.2017.09.007, PMID: 28988769 PMC5687509

[17] Jiang Q Fu Q Chang Y Liu Z Zhang J Xu L . CD19+ tumor-infiltrating B-cells prime CD4+ T-cell immunity and predict platinum-based chemotherapy efficacy in muscle-invasive bladder cancer. Cancer Immunol Immunother. (2019) 68:45–56. doi: 10.1007/s00262-018-2250-9, PMID: 30259082 PMC11028136

[18] Song D Powles T Shi L Zhang L Ingersoll MA Lu YJ . Bladder cancer, a unique model to understand cancer immunity and develop immunotherapy approaches. J Pathol. (2019) 249:151–65. doi: 10.1002/path.5306, PMID: 31102277 PMC6790662

[19] Liu Z Zhou Q Wang Z Zhang H Zeng H Huang Q . Intratumoral TIGIT+ CD8+ T-cell infiltration determines poor prognosis and immune evasion in patients with muscle-invasive bladder cancer. J Immunother Cancer. (2020) 8:e000978. doi: 10.1136/jitc-2020-000978, PMID: 32817209 PMC7430558

[20] Attalla K Farkas AM Anastos H Audenet F Galsky MD Bhardwaj N . TIM-3 and TIGIT are possible immune checkpoint targets in patients with bladder cancer. Urol Oncol. (2022) 40(9):403–6. doi: 10.1016/j.urolonc.2020.06.007, PMID: 32665122 PMC7980780

[21] Zeng H Zhou Q Wang Z Zhang H Liu Z Huang Q . Stromal LAG-3 + cells infiltration defines poor prognosis subtype muscle-invasive bladder cancer with immunoevasive contexture. J Immunother Cancer. (2020) 8:e000651. doi: 10.1136/jitc-2020-000651, PMID: 32540859 PMC7295439

[22] Thommen DS Schumacher TN . T cell dysfunction in cancer. Cancer Cell. (2018) 33:547–62. doi: 10.1016/j.ccell.2018.03.012, PMID: 29634943 PMC7116508

[23] Anderson AC Joller N Kuchroo VK . Lag-3, Tim-3, and TIGIT: Co-inhibitory receptors with specialized functions in immune regulation. Immunity. (2016) 44:989–1004. doi: 10.1016/j.immuni.2016.05.001, PMID: 27192565 PMC4942846

[24] Kong Y Zhu L Schell TD Zhang J Claxton DF Ehmann WC . T-Cell immunoglobulin and ITIM domain (TIGIT) associates with CD8+ T-cell exhaustion and poor clinical outcome in AML patients. Clin Cancer Res. (2016) 22:3057–66. doi: 10.1158/1078-0432.CCR-15-2626, PMID: 26763253

[25] Chauvin J-M Pagliano O Fourcade J Sun Z Wang H Sander C . TIGIT and PD-1 impair tumor antigen-specific CD8^+^ T cells in melanoma patients. J Clin Invest. (2015) 125:2046–58. doi: 10.1172/JCI80445, PMID: 25866972 PMC4463210

[26] Zhang Q Bi J Zheng X Chen Y Wang H Wu W . Blockade of the checkpoint receptor TIGIT prevents NK cell exhaustion and elicits potent anti-tumor immunity. Nat Immunol. (2018) 19:723–32. doi: 10.1038/s41590-018-0132-0, PMID: 29915296

[27] Harjunpaa H Guillerey C . TIGIT as an emerging immune checkpoint. Clin Exp Immunol. (2020) 200:108–19. doi: 10.1111/cei.13407, PMID: 31828774 PMC7160651

[28] Solomon BL Garrido-Laguna I . TIGIT: a novel immunotherapy target moving from bench to bedside. Cancer Immunol Immunother. (2018) 67:1659–67. doi: 10.1007/s00262-018-2246-5, PMID: 30232519 PMC11028339

[29] Chocarro L Blanco E Zuazo M Arasanz H Bocanegra A Fernandez-Rubio L . Understanding LAG-3 signaling. Int J Mol Sci. (2021) 22:5282. doi: 10.3390/ijms22105282, PMID: 34067904 PMC8156499

[30] Andrews LP Marciscano AE Drake CG Vignali DA . LAG3 (CD223) as a cancer immunotherapy target. Immunol Rev. (2017) 276:80–96. doi: 10.1111/imr.12519, PMID: 28258692 PMC5338468

[31] Burugu S Gao D Leung S Chia SK Nielsen TO . LAG-3+ tumor infiltrating lymphocytes in breast cancer: clinical correlates and association with PD-1/PD-L1+ tumors. Ann Oncol. (2017) 28:2977–84. doi: 10.1093/annonc/mdx557, PMID: 29045526

[32] Fucikova J Rakova J Hensler M Kasikova L Belicova L Hladikova K . Tim-3 dictates functional orientation of the immune infiltrate in ovarian cancer. Clin Cancer Res. (2019) 25:4820–31. doi: 10.1158/1078-0432.CCR-18-4175, PMID: 31076549

[33] He Y Yu H Rozeboom L Rivard CJ Ellison K Dziadziuszko R . LAG-3 protein expression in non-small cell lung cancer and its relationship with PD-1/PD-L1 and tumor-infiltrating lymphocytes. J Thorac Oncol. (2017) 12:814–23. doi: 10.1016/j.jtho.2017.01.019, PMID: 28132868

[34] Rotte A Jin JY Lemaire V . Mechanistic overview of immune checkpoints to support the rational design of their combinations in cancer immunotherapy. Ann Oncol. (2018) 29:71–83. doi: 10.1093/annonc/mdx686, PMID: 29069302

[35] Tran L Xiao JF Agarwal N Duex JE Theodorescu D . Advances in bladder cancer biology and therapy. Nat Rev Cancer. (2021) 21:104–21. doi: 10.1038/s41568-020-00313-1, PMID: 33268841 PMC10112195

[36] Patel VG Oh WK Galsky MD . Treatment of muscle-invasive and advanced bladder cancer in 2020. CA Cancer J Clin. (2020) 70:404–23. doi: 10.3322/caac.21631, PMID: 32767764

[37] Crispen PL Kusmartsev S . Mechanisms of immune evasion in bladder cancer. Cancer Immunol Immunother. (2020) 69:3–14. doi: 10.1007/s00262-019-02443-4, PMID: 31811337 PMC6949323

[38] Vaithegi R Pai K Calicut Kini Rao A Vidya M Swathi P Nischitha S . Clinicopathological study and molecular subtyping of muscle-invasive bladder cancer (MIBC) using dual immunohistochemical (IHC) markers. Diagn Pathol. (2025) 20:10. doi: 10.1186/s13000-025-01603-8, PMID: 39856762 PMC11759442

[39] Ren X Guo A Geng J Chen Y Wang X Zhou L . Pan-cancer analysis of co-inhibitory molecules revealing their potential prognostic and clinical values in immunotherapy. Front Immunol. (2025) 16:1544104. doi: 10.3389/fimmu.2025.1544104, PMID: 40196117 PMC11973099

[40] Tavana S Mokhtari Z Sanei MH Heidari Z Dehghanian AR Faghih Z . Clinicopathological significance and prognostic role of LAG3 + tumor-infiltrating lymphocytes in colorectal cancer; relationship with sidedness. Cancer Cell Int. (2023) 23:23. doi: 10.1186/s12935-023-02864-3, PMID: 36765348 PMC9912542

[41] Dadhania V Zhang M Zhang L Bondaruk J Majewski T Siefker-Radtke A . Meta-analysis of the luminal and basal subtypes of bladder cancer and the identification of signature immunohistochemical markers for clinical use. EBioMedicine. (2016) 12:105–17. doi: 10.1016/j.ebiom.2016.08.036, PMID: 27612592 PMC5078592

[42] Hodgson A Liu SK Vesprini D Xu B Downes MR . Basal-subtype bladder tumours show a ‘hot’ immunophenotype. Histopathology. (2018) 73:748–57. doi: 10.1111/his.13696, PMID: 29947424

[43] Mo Q Nikolos F Chen F Tramel Z Lee YC Hayashi K . Prognostic power of a tumor differentiation gene signature for bladder urothelial carcinomas. Natl Cancer Inst. (2019) 111:1236. doi: 10.1093/jnci/djx243, PMID: 29342309 PMC6279371

[44] Wankowicz S Werner L Orsola A Novak J Bowden M Choueiri TK . Differential Expression of PD-L1 in High Grade T1 vs Muscle Invasive Bladder Carcinoma and its Prognostic Implications. J. Urol. (2017) 198:817–23. doi: 10.1016/j.juro.2017.04.102, PMID: 28487100

[45] Boormans JL Zwarthoff EC Black PC Goebell PJ Kamat AM Nawroth R . New horizons in bladder cancer research. Urol Oncol. (2020) 38:867–85. doi: 10.1016/j.urolonc.2018.12.014, PMID: 30852032

[46] Khandakar H Kaushal S Seth A Sahoo RK Narwal A Jangir H . Comparative evaluation of PD-L1 expression and tumor immune microenvironment in molecular subtypes of muscle-invasive bladder cancer and its correlation with survival outcomes. Am J Clin Pathol. (2025) 163:708–22. doi: 10.1093/ajcp/aqae176, PMID: 39805149

[47] Jin S Shang Z Wang W Gu C Wei Y Zhu Y . Immune co-inhibitory receptors CTLA-4, PD-1, TIGIT, LAG-3, and TIM-3 in upper tract urothelial carcinomas: A large cohort study. J Immunother. (2023) 46:154–59. doi: 10.1097/CJI.0000000000000466, PMID: 37017991 PMC10072209

[48] Wu K Zeng J Shi X Xie J Li Y Zheng H . Targeting TIGIT inhibits bladder cancer metastasis through suppressing IL-32. Front Pharmacol. (2022) 12:801493. doi: 10.3389/fphar.2021.801493, PMID: 35069212 PMC8766971

[49] Liu Z Zeng H Jin K Yu Y You R Zhang H . TIGIT and PD-1 expression atlas predicts response to adjuvant chemotherapy and PD-L1 blockade in muscle-invasive bladder cancer. Br J Cancer. (2022) 126:1310–17. doi: 10.1038/s41416-022-01703-y, PMID: 35039625 PMC9042924

[50] Chu X Tian W Wang Z Zhang J Zhou R . Co-inhibition of TIGIT and PD-1/PD-L1 in cancer immunotherapy: mechanisms and clinical trials. Mol Cancer. (2023) 22:93. doi: 10.1186/s12943-023-01800-3, PMID: 37291608 PMC10249258

[51] Cano Barbadilla T Álvarez Pérez M Prieto Cuadra JD Dawid de Vera MT Alberca-del Arco F García Muñoz I . The role of immunohistochemistry as a surrogate marker in molecular subtyping and classification of bladder cancer. Diagnostics. (2024) 14:2501. doi: 10.3390/diagnostics14222501, PMID: 39594166 PMC11592502

[52] Schwarzova L Varchulova Novakova Z Danisovic L Ziaran S . Molecular classification of urothelial bladder carcinoma. Mol Biol Rep. (2023) 50:7867–77. doi: 10.1007/s11033-023-08689-7, PMID: 37525073 PMC10460735

[53] van Dorp J van der Heijden MS . The bladder cancer immune micro-environment in the context of response to immune checkpoint inhibition. Front Immunol. (2023) 14:1235884. doi: 10.3389/fimmu.2023.1235884, PMID: 37727793 PMC10505825

[54] Kong X Zhang J Chen S Wang X Xi Q Shen H . Immune checkpoint inhibitors: breakthroughs in cancer treatment. Cancer Biol Med. (2024) 21:451–72. doi: 10.20892/j.issn.2095-3941.2024.0055, PMID: 38801082 PMC11208906

[55] Ledderose S Ledderose C Ledderose GJ . Expression of immune checkpoint molecules TIGIT and TIM-3 by tumor-infiltrating lymphocytes predicts poor outcome in sinonasal mucosal melanoma. Pathol Res Pract. (2024) 260:155468. doi: 10.1016/j.prp.2024.155468, PMID: 39018929

[56] Cai L Li Y Tan J Xu L Li Y . Targeting LAG-3, TIM-3, and TIGIT for cancer immunotherapy. J Hematol Oncol. (2023) 16:101. doi: 10.1186/s13045-023-01499-1, PMID: 37670328 PMC10478462

[57] Cillo AR Cardello C Shan F Karapetyan L Kunning S Sander C . Blockade of LAG-3 and PD-1 leads to co-expression of cytotoxic and exhaustion gene modules in CD8^+^ T cells to promote antitumor immunity. Cell. (2024) 187:4373–88.e15. doi: 10.1016/j.cell.2024.06.036, PMID: 39121849 PMC11346583

